# Effects of weifuchun tablet for chronic atrophic gastritis: A protocol for systematic review and meta-analysis

**DOI:** 10.1097/MD.0000000000020374

**Published:** 2020-05-29

**Authors:** Liangjun Yang, Zhipeng Hu, Jiajie Zhu, Baoying Fei

**Affiliations:** a Department of Gastroenterology, Tongde Hospital of Zhejiang Province; b Hospital of Chengdu University of Traditional Chinese Medicine, Sichuan Province; c Digestive Disease Institute of Integrated Traditional Chinese and Western Medicine, Zhejiang Academy of Traditional Chinese Medicine, Zhejiang Province, China.

**Keywords:** chronic atrophic gastritis, protocol, systematic review and meta-analysis, weifuchun

## Abstract

**Background:**

Chronic atrophic gastritis (CAG) is defined as an important precancerous disease in the development of gastric cancer. Early intervention of CAG is of great significance in reducing symptoms and blocking its progression to gastric cancer. Weifuchun (WFC) tablet is a classic Chinese patent medicine used to treat CAG. However, there is no systematic review related to WFC for atrophic gastritis published in English. we will conduct systematic review and meta-analysis to provide more evidence on the effectiveness and safety for clinical use of WFC.

**Methods and analysis:**

Three English database and 4 Chinese databases will be searched from its inception to April 2020. Two trained researchers will independently select the qualified studies for data extraction and assess the quality and risk of bias. Then the meta-analyses will be performed by using the RevMan 5.2 and stata 14.0. The heterogeneity of data will be investigated by Cochrane X^2^ and *I*^*2*^ tests. Sensitivity analysis will be conducted to evaluate the stability of the results. A funnel plot analysis and Egger's test will be drawn to assess the publication bias. Finally, we will use the Grading of Recommendations Assessment, Development and Evaluate system to evaluate the methodological quality.

**Results:**

The results of our research will be published in a peer-reviewed journal.

**Conclusion:**

The conclusion of our systematic review will provide evidence to judge whether WFC is an effective intervention for patient with CAG.

**OSF registration number:**

10.17605/OSF.IO/2UTMB

## 1 Introduction

Gastric cancer is 1 of the most common and aggressive tumors in the digestive system around the world, and remains the most prevalent cancer in Eastern Asia, especially in China.^[1]^ According to the “Correa cascade”, the occurrence of gastric cancer is a continuous multi-stage biological process starting from chronic superficial gastritis, atrophic gastritis, and finally to gastric adenocarcinoma.^[2]^ Chronic atrophic gastritis (CAG) is a well-defined histopathological entity characterized by chronic inflammation of the gastric mucosa accompanied by the loss of gastric gland cells and replaced by intestinal epithelium and fibrous tissue. It is usually defined as an important precancerous disease in the development of gastric cancer.^[3]^ Based on a multi-center national study in China, CAG accounted for 25.8% of patients with gastritis.^[4]^ Therefore, early intervention of this disease is imperative to prevent progression to gastric cancer

Currently, *Helicobacter pylori* eradication, acid suppression, and vitamin supplement are commonly used in the treatment of CAG. However, these therapeutic measures have several limitations and side effects,^[5]^ which restricts the treatment of CAG. Traditional Chinese medicine is wildly used in China for its multi-target and fewer side effects, and has been used to treat CAG.^[6]^ Weifuchun (WFC) tablet is a classic Chinese patent medicine composed of *Panax ginseng*, *Isodon amethystoide*s, and *Bran Fried hovenia dulcis*. It is wildly used in digestive diseases by strengthening the spleen, replenishing qi, and invigorating bloodm and has been proved by the China Food and Drug Administration for the treatment of chronic gastritis (approved No. Z20040003). More and more clinical studies have reported the application of WFC for better efficacy in patients with CAG.^[7,8]^ According the consensus on the diagnosis and treatment of integrated Chinese and Western medicine for CAG in 2017, WFC was recommended as an alternative treatment for CAG.^[9]^ However, the consensus did not grade the quality level of evidence of this patent medicine. A systematic review is urgently needed to support the effectiveness and safety of WFC. In this work, we will systematically evaluate the clinical efficacy of WFC for CAG using a meta-analysis method, so as to provide a reliable evidence for clinical practice.

## 2 Methods and analysis

### 2.1 Study registration

This work has been registered at Open Science Framework (OSF, https://osf.io/), an open source project management that helps in the design of studies. The registration DOI of this study is 10.17605/OSF.IO/2UTMB. The protocol of our meta-analysis is reported followed the guideline of the Preferred Reporting Items for Systematic Review and Meta-Analysis Protocols recommendations.^[10]^

### 2.2 Inclusion and exclusion criteria

#### 2.2.1 Study design

Regardless of the blind method and language, the design types of studies included in this work will be limited to randomized controlled trial (RCT), while cross-over studies will be excluded in the research. Non-randomized control studies and observational study will not be eligible for inclusion in the review.

#### 2.2.2 Type of participants

All patients who are diagnosed with CAG by endoscopic assessment and mucosal biopsy. There will be no limitation about age, gender, region, and other factors.

#### 2.2.3 Interventions/Comparators

Those studies which set WFC alone for the treatment of CAG will be included in our research. Studies that combine WFC with other treatment as experimental therapies will be excluded. Control therapy can be any type of conventional medications, including folic acid, vitacoenzyme, and Chinese herbal compound. The treatment durations should be at least 12 weeks.

#### 2.2.4 Outcomes

The primary outcomes of this review will focus on the improvement of gastric histopathology. The secondary outcomes included the improvement of clinical symptoms, endoscopic improvement, *Helicobacter pylori* eradication rate and so on. Any adverse events will also be included in the work.

### 2.3 Study search

To ascertain the relevant literature, three English databases including PubMed, Embase, Cochrane Library Central Register of Controlled Trials and 4 Chinese databases including China National Knowledge Infrastructure database, Wanfang Data Knowledge Service Platform, the VIP information resource integration service platform, China Biology Medicine Disc will be searched from its inception to April 2020. In addition, Google scholar, Bing scholar, and Baidu scholar will be will retrieved to find out other related literature. English and Chinese were applied as language restrictions. Moreover, the Chinese Clinical Trial Registry and ClinicalTrials.gov will also be searched. Two authors (Liangjun Yang and Zhipeng Hu) will search and screen all the citations independently.

A search strategy that combines MeSH terms and free words will be adopted. The search strategy was as follows:

(1) 1: Search ((((((atrophic gastritis[MeSH Terms]) OR gastritis, atrophic[MeSH Terms]) OR CAG [Title/Abstract]) OR chronic gastritis[Title/Abstract]) OR gastric atrophy[Title/Abstract]) OR gastric mucosal atrophy[Title/Abstract]) OR CAG[Title/Abstract]
(2) 2: Search ((weifuchun[Title/Abstract]) OR weifuchun tablet[Title/Abstract]) OR weifuchun pian[Title/Abstract]
(3) 3: Search (((((((((randomized controlled trial[Title/Abstract]) OR RCT[Title/Abstract]) OR random[Title/Abstract]) OR randomly[Title/Abstract]) OR random allocation[Title/Abstract]) OR allocation[Title/Abstract]) OR randomized control trial[Title/Abstract]) OR controlled clinical trial[Title/Abstract]) OR clinical trial[Title/Abstract]) OR clinical study[Title/Abstract]

#1 and #2 and #3

### 2.4 Study selection

**Figure 1:**
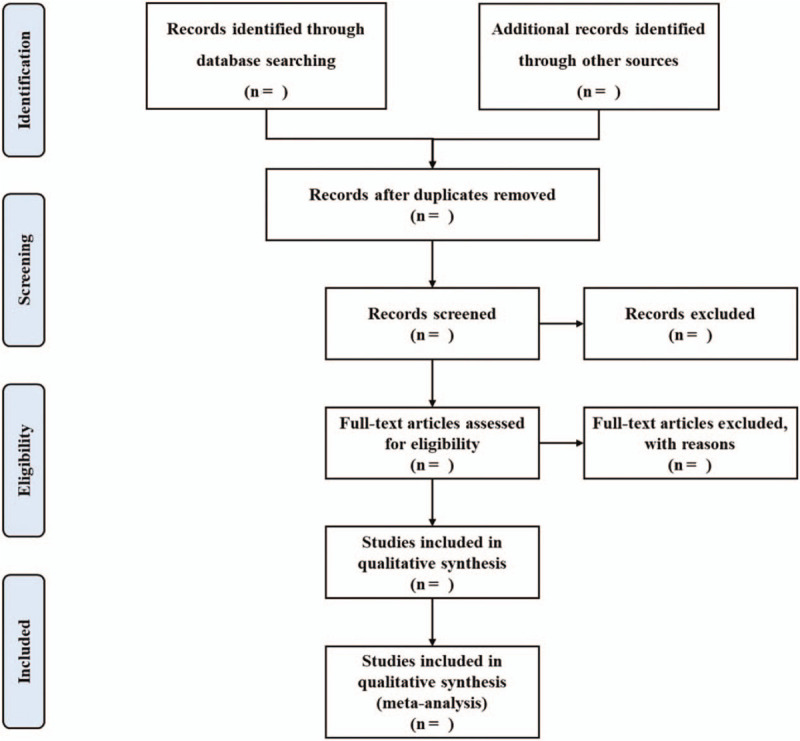
Flow chart of study selection.

The electronic citations extracted out from the above databases will be managed by EndNote X9.0 (Stanford, Connecticut, https://endnote.com). Two methodological trained reviewers (Liangjun Yang and Zhipeng Hu) will independently evaluate the titles and abstracts of the searched studies and screen the citations in accordance with the established selection criteria. By reading the full text, those articles that meet the criteria will be further determined. Any discrepancies generated between the 2 reviewers will be resolved through discussion. A Preferred Reporting Items for Systematic Reviews and Meta-analysis flow chart will be drawn to illustrate the study selection procedure (Fig. 1).

### 2.5 Data extraction and management

The results of the data extraction will be checked, and the data of those qualified articles will be export to Microsoft Excel. Two researchers will extract information from the studies that met the inclusion criteria, including the first authors of the article, year of publication, pathological type of gastric, interventions in experimental group and control group, time of treatment, ample size in each group, age, gender, outcome indicators, and adverse events. When data are missing, or unclear, we will contact the corresponding author for more detailed information. If the methodological details are not described in papers, we will contact the author for more explanation.

### 2.6 Assessment of risk of bias in included studies

The Cochrane collaboration's tool, an established and reliable tool for assessing the risk of bias, will be used in studies. Two reviewers will evaluate the methodological quality of the included trials independently. In this tool, the risk of bias of a trial is evaluated through 7 items, include random sequence generation (selection bias), allocation concealment (selection bias), blinding of participants and personnel (performance bias), blinding of outcome assessment (detection bias), incomplete outcome data (attrition bias), selective reporting (reporting bias), other bias. The assessment will be classified as “Low risk”, “High risk” or “Unclear risk”. Inconsistencies between the 2 reviewers will be resolved by discussion of all reviewers.

### 2.7 Data analysis

RevMan 5.2 (Cochrane, London, UK) and stata 14.0 software will be applied to create forest plots and conduct subgroup analysis and sensitivity analysis. For dichotomous variables, the effect size will be represented as risk ratio and 95% confidence interval. In addition, a mantel-haenszel method will be employed to calculate them and the odds ratio (OR) will be applied to analyze. For continuous variables, the effect size will be represented as mean difference and 95% confidence interval. Statistical heterogeneity will be computed by Cochrane *X*^*2*^ and *I*^*2*^ tests.^[11]^ If *P*≥.05 and *I*^2^≤50%, it suggests that heterogeneity is not important and the differences between them can be ignored. If *P* < .05 and *I*^*2*^ > 50%, it manifests that the study has significant statistic heterogeneity. If there is no statistical heterogeneity between the results of each study, the fixed effect model will be applied. If there is statistical heterogeneity among the results of each study, the random effect model will be used. The results will be displayed in tables and figures when the quantitative synthesis is not suitable.

### 2.8 Assessment of heterogeneity

If there is substantial heterogeneity between studies, subgroup analysis, meta-regression, or descriptive analysis will be conducted for heterogeneity. There are 3 kinds of hypotheses for subgroup analysis: disease status at baseline, duration of intervention, and type of concomitant medication.^[12]^ The subgroup analysis will be performed based on subgroup assumptions. We will evaluate the credibility of the subgroup analysis in term of the guidance.^[13]^ If there are enough researches, meta-regression will be performed to clarify the source of heterogeneity.

### 2.9 Sensitivity analysis

To investigate the robustness of the results, a sensitivity analysis will be performed for the outcomes. We will exclude each study included in the analysis 1 by 1. Then we will re-analyze and pool the data and compare the difference between the re-obtained effects and the original effects. In this way, we will be able to assess whether these factors affect the results and whether the results are robust.

### 2.10 Assessment of reporting biases

When more than ten studies are included, a funnel plot will be drawn to assess the publication bias. Through using the Egger's test, publication bias will be statistical appraised.^[14]^*P* < .05 is considered to have publication bias.

### 2.11 Grading the quality of evidence

The Grading of Recommendations Assessment, Development and Evaluation, a widely used tool in evaluating the quality of assessment,^[15]^ will be applied to assess the quality of evidence for the main outcomes. The quality of evidence will be assorted into “high”, “moderate”, “low”, and “very low” quality.

### 2.12 Patient and public involvement

Patient and public were not involved in this study.

### 2.13 Ethics and dissemination

This systematic review will not need ethical approval because the data used are not linked to individual patient. This study comprehensively evaluates the existing research evidence of WFC in the treatment of CAG and will provide clinicians with evidence-based medical support. The results of this review will be spread by being published in a peer-reviewed journal.

## 3 Discussion

CAG is an important precursor lesion of gastric carcinogenesis, a major health problem with high morbidity and mortality in China.^[16]^ Therefore, early attention and intervention should be conducted in the management of atrophic gastritis. WFC is a famous Chinese patent medicine for treating chronic gastritis in clinical practice, and a series of clinical studies have been conducted on it. However, there is no systematic review related to WFC for CAG published in English, which limits the clinical application of this patent. In this study, we will conduct systematic review and meta-analysis to provide more evidence on the effectiveness and safety for clinical use of WFC. These findings may help provide guidance to clinicians in the treatment of CAG.

## Author contributions

**Conceptualization:** Liangjun Yang, Zhipeng Hu.

**Data curation:** Liangjun Yang, Zhipeng Hu.

**Formal analysis:** Jiajie Zhu

**Funding acquisition:** Baoying Fei.

**Investigation:** Liangjun Yang, Zhipeng Hu.

**Methodology:** Liangjun Yang, Zhipeng Hu, Jiajie Zhu.

**Project administration:** Baoying Fei.

**Resources:** Liangjun Yang, Zhipeng Hu, Jiajie Zhu.

**Software:** Liangjun Yang, Zhipeng Hu.

**Supervision:** Baoying Fei.

**Writing – original draft:** Liangjun Yang.

**Writing – review & editing:** Zhipeng Hu, Jiajie Zhu.

## References

[1] Ferlay J Soerjomataram I Dikshit R. Cancer incidence and mortality worldwide: Sources, methods and major patterns in GLOBOCAN 2012: Globocan 2012. Int J Cancer 2015;136:E359–86. 25220842 10.1002/ijc.29210

[2] Correa P. A human model of gastric carcinogenesis. Cancer Res 1988;48:3554–60. 3288329

[3] Ohata H Kitauchi S Yoshimura N. Progression of chronic atrophic gastritis associated with Helicobacter pylori infection increases risk of gastric cancer. Int J Cancer 2004;109:138–43. 14735480 10.1002/ijc.11680

[4] Du Y Bai Y Xie P. Chronic gastritis in China: a national multi-center survey. BMC Gastroenterol 2014;14:21. 24502423 10.1186/1471-230X-14-21 PMC3922313

[5] den Hollander WJ Kuipers EJ. Current pharmacotherapy options for gastritis. Expert Opin Pharmacother 2012;13:2625–36. 23167300 10.1517/14656566.2012.747510

[6] Dai Y-K Zhang Y-Z Li D-Y. The efficacy of Jianpi Yiqi therapy for chronic atrophic gastritis: A systematic review and meta-analysis. PLoS One 2017;12:e0181906. 28738092 10.1371/journal.pone.0181906 PMC5524332

[7] Shu M Jiang Z Wang Y. Clinical efficacy of weifuchun combined with retinoic acid on treatment of patients with PLGC and effect on expressions of gene Rb and C-erbB-2. Biomedical Research 2017;0:606–9.

[8] Li H-Z Wang H Wang G-Q. Treatment of gastric precancerous lesions with Weiansan. World J Gastroenterol 2006;12:5389–92. 16981274 10.3748/wjg.v12.i33.5389 PMC4088211

[9] Junxiang L Jing C Bin L. Consensus on the diagnosis and treatment of integrated Chinese and Western medicine for chronic atrophic gastritis. Chinese Journal of Integrated Traditional and Western Medicine on Digestion 2018;26:121–31.

[10] Moher D Shamseer L Clarke M. Preferred reporting items for systematic review and meta-analysis protocols (PRISMA-P) 2015 statement. Syst Rev 2015;4:1. 25554246 10.1186/2046-4053-4-1 PMC4320440

[11] Higgins JPT Thompson SG. Quantifying heterogeneity in a meta-analysis. Stat Med 2002;21:1539–58. 12111919 10.1002/sim.1186

[12] Oxman AD Guyatt GH. A consumer's guide to subgroup analyses. Ann Intern Med 1992;116:78–84. 1530753 10.7326/0003-4819-116-1-78

[13] Sun X Briel M Walter SD. Is a subgroup effect believable? Updating criteria to evaluate the credibility of subgroup analyses. BMJ 2010;340:c117. 20354011 10.1136/bmj.c117

[14] Peters JL Sutton AJ Jones DR. Contour-enhanced meta-analysis funnel plots help distinguish publication bias from other causes of asymmetry. J Clin Epidemiol 2008;61:991–6. 18538991 10.1016/j.jclinepi.2007.11.010

[15] Group GW. Grading quality of evidence and strength of recommendations. BMJ 2004;328:1490. 15205295 10.1136/bmj.328.7454.1490 PMC428525

[16] Gao K Wu J. National trend of gastric cancer mortality in China (2003-2015): a population-based study. Cancer Commun (Lond) 2019;39:24. 31046840 10.1186/s40880-019-0372-x PMC6498569

